# RBFNN Design Based on Modified Nearest Neighbor Clustering Algorithm for Path Tracking Control

**DOI:** 10.3390/s21248349

**Published:** 2021-12-14

**Authors:** Dongxi Zheng, Wonsuk Jung, Sunghoon Kim

**Affiliations:** 1 Department of Electronics Convergence Engineering, Wonkwang University, Iksan 54538, Korea; zhengdongxi1979@wku.ac.kr; 2 School of Mechanical and Intelligent Manufacturing, Jiujiang University, Jiujiang 332005, China; 3 School of Mechanical Engineering, Chungnam National University, Daejeon 34134, Korea; 4 Wonkwang Institute of Material Science and Technology, Wonkwang University, Iksan 54538, Korea

**Keywords:** radial basis function neural network (RBFNN), nearest neighbor-based clustering (MNNC), sample optimization, path tracking

## Abstract

Radial basis function neural networks are a widely used type of artificial neural network. The number and centers of basis functions directly affect the accuracy and speed of radial basis function neural networks. Many studies use supervised learning algorithms to obtain these parameters, but this leads to more parameters that need to be determined, thereby making the system more complex. This study proposes a modified nearest neighbor-based clustering algorithm for training radial basis function neural networks. The calculation of this clustering algorithm is not large, and it can adapt to varying densities. Furthermore, it does not require researchers to set parameters based on experience. Simulation proves that the clustering algorithm can effectively cluster samples and optimize the abnormal samples. The radial basis function neural network based on modified nearest neighbor-based clustering has higher accuracy in curve fitting than the conventional radial basis function neural network. Finally, the path tracking control based on a radial basis function neural network of a magnetic microrobot is investigated, and its effectiveness is verified through simulation. The test accuracy and training accuracy of the radial basis function neural network was improved by 23.5% and 7.5%, respectively.

## 1. Introduction

Path tracking control is a commonly used motion control method for vehicles and robots. Owing to its simple structure, easy operation and adjustment, and robustness, the proportion integral differential (PID, as shown in Appendix A) controller is often used for path tracking control [1,2]. However, the ability of PID in dealing with nonlinear systems is limited. Therefore, fuzzy PID control was developed. B.B. Ghosh et al. developed a fuzzy-PID-based controller to control the two degrees of freedom parallel manipulator. The control system has almost no overshoot based on the fuzzy-PID [3]. J.A. Algarin-Pinto et al. compared the fuzzy-PID with general PID for path tracking control of biomimetic autonomous underwater vehicles. The experiment results showed that path tracking control error with general PID was over 9%, but with fuzzy-PID was less than 2% [4]. T.A. Mai et al. applied fuzzy PID in path-following control of a nonholonomous mobile robot. Under the control system based on fuzzy-PID, the distance error of path-following control could be reduced from 0.172 m to 0.041 m [5]. Nonetheless, fuzzy rules require strong prior knowledge. Due to the time-varying dynamics, nonlinear uncertainty of the control object, and environmental interference, it is extremely difficult to control the high-precision path tracking for the linear state observer because it is difficult for the linear state observer to compensate errors of the nonlinear system [6]. The previous methods are incapable of addressing these issues. Although a sliding mode controller could control the trajectory tracking of a nonlinear system [7,8], it occasionally caused a large lateral acceleration in the trajectory tracking using the sliding mode control method. 

To deal with nonlinear systems, C. Liu et al. proposed a nonlinear adaptive controller based on PID [9]. B. Smeresky et al. discussed a deterministic artificial intelligence-instantiated method for a nonlinear system, which stems from a lineage of nonlinear adaptive control [10]. Compared with these methods, artificial neural networks have attracted increasing interest from researchers because they do not require complex modeling process or powerful processing, and have adaptive capabilities in constantly changing and noisy environments. Utilizing the learning ability of an artificial neural network facilitates improved flexibility of controller design, particularly when the dynamics of the controlled object are complex and highly non-linear [11]. Radial basis function neural networks (RBFNN) have the advantages of fast learning convergence speed and strong approximation ability; they have been used in finite-time trajectory tracking control of n-link robotic manipulators [12], longitudinal speed tracking of autonomous vehicles [13], trajectory tracking for a robotic helicopter [14], and tracking control of a nonholonomic wheel-legged robot in complex environments [15]. In these cases, the control systems based on RBFNN showed good accuracy and stability.

Before running an RBFNN, it is necessary to determine the relevant parameters, such as the type and number of basis functions, the center and the width of the basis functions, and the weight of each hidden layer neuron. These parameters affect not only the learning time, but also the controller performance [16,17]. To optimize the relevant parameters of an RBFNN, supervised learning or unsupervised learning methods can be used. In supervised learning, other intelligent algorithms are introduced to optimize the parameters of the RBFNN. F. Fernandez-Navarro et al. investigated performance of an RBFNN based on support vector machines (VSM). The parameters of VSM should be defined [18]. H.C. Huang et al. presented an evolutionary radial basis function neural network with genetic algorithm (GA) and artificial immune system (AIS) for tracking control of autonomous robots. Although the controller based on a GAAIS-RBFNN showed better performance than the controller based on an individual genetic algorithm and artificial immune system, GAAIS-RBFNN involved more variables to be decided [19]. Z.Y. Chen et al. trained the RBFNN by particle swarm optimization and genetic algorithm. The RBFNN showed good learning performance, but the algorithm was more complex [20]. When using unsupervised learning to design and optimize the parameters of an RBFNN, the clustering algorithm is a commonly used method that can speedily converge and avoid overfitting. A. Guillén et al. developed a clustering algorithm with a possibilistic partition to get the initial center of hidden layer neurons of an RBFNN. The algorithm showed better robustness than other general RBFNNs [21]. S.K. Oh et al. applied a k-means clustering algorithm in setting the center of hidden layer neurons of RBFNN; the algorithm showed good accuracy [22]. C.C. Liao et al. introduced an RBFNN-based control system for tracking the maximum power point of a photovoltaic system. The parameters of RBFNN were determined by the modified k-means clustering algorithm. The experiment results proved the tracking method was effective [23]. 

However, most clustering algorithms need to determine some parameters in advance; for example, k-means requires the number of clusters and initial center of cluster to cluster the samples; density-based spatial clustering of applications with noise (DBSCAN) requires the radius of the scan and the minimum number of samples of the cluster for clustering; clustering by fast search and find of density peaks clustering (DPC) requires the threshold of distance. These parameters are extremely important and affect the results of clustering significantly, but it is necessary for users to determine and adjust the parameters based on experience, which is difficult. Moreover, common clustering algorithms often require iteration or a large calculation that reduces the efficiency of the clustering algorithm. To avoid these issues, we propose a modified nearest neighbor-based clustering (MNNC) algorithm according to the characteristics of curve fitting and path following control datasets. Unlike other clustering algorithms, MNNC clusters the samples referring to the distance between the sample and its nearest neighbor. It is easy to utilize this algorithm because it requires fewer parameters to be determined. Furthermore, MNNC improves the approach to searching for neighbors, thus it does not require iteration and requires less calculation which increases the efficiency of clustering. We evaluated the clustering results using accuracy (ACC) and adjusted Rand index (ARI); the simulation results show that ACC and ARI using MNNC are 20% and 10% higher than the common clustering algorithms, respectively. MNNC can also detect and optimize the outlier samples, and the simulation results show that the optimization of outlier samples can decrease the curve fitting errors by 10–50%. MNNC was used to set the initial parameters of RBFNN that can automatically adjust the number of hidden layer nodes according to the accuracy requirements. In particular, we applied the proposed method to a path tracking simulation of a spiral-type magnetic microrobot to generate a rotating magnetic field (RMF) to reach the desired position. Consequently, the proposed method featured 20 % lower error than conventional RBFNN.

The remainder of this study is organized as follows. Section 2 introduces the concept of RBFNN based on MNNC for path tracking. Section 3 describes a novel clustering algorithm that is applied in optimizing the samples in Section 4. Section 5 introduces the control system for the path tracking of magnetic microrobot and develops MNNC to train RBFNN for path tacking. Finally, the discussion and conclusion are presented in Section 6 and Section 7, respectively.

## 2. Concept of RBFNN Algorithm Based on MNNC for Path Tracking

**Figure 1:**
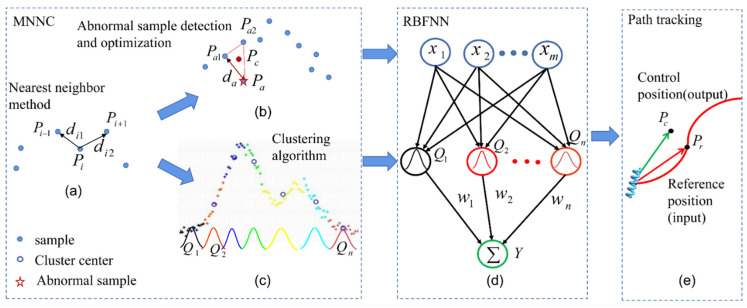
Concept of an RBFNN algorithm based on MNNC for path tracking: (**a**) calculation of the distance to obtain the nearest neighbor; (**b**) detection of the abnormal samples based on nearest neighbor method; (**c**) clustering of the samples based on the nearest neighbor; (**d**) construction of the RBFNN based on MNNC; (**e**) obtaining the reference position by the MNNC-based RBFNN in the path tracking system.

Figure 1 illustrates the proposed RBFNN algorithm based on MNNC for path tracking. The entire algorithm consists of three parts: MNNC, RBFNN, and path tracking. The MNNC is used to obtain the initial parameter of RBFNN and optimize the training samples of RBFNN. First, MNNC characterizes sample *P_i_* by distance (*d_i_*, more variables are shown in Appendix B) between *P_i_* and its nearest neighbor distance and classifies samples with similar *d_i_* into one class. The datasets of curve-fitting and path-following control have obvious temporal or spatial order characteristics; when searching for the nearest neighbor, the search range can be reduced by improving the searching direction in order to reduce the calculation of the clustering algorithm, as shown in Figure 1a, where *d_i_* is the minimal value of *d_i_*_1_ and *d_i_*_2_. Thereafter, the MNNC can detect and optimize the abnormal samples, as shown in Figure 1b. *P_a_* is defined as an abnormal sample because *P_a_* corresponds to the longest distance *d_a_*, and there are no similar samples around *P_a_*. We construct a triangle by *P_a_*, *P_a_*_1_, and *P_a_*_2_, where *P_a_*_1_ and *P_a_*_2_ are the neighbors of *P_a_*. Next, we obtain *P_c_*, which is the center of the triangle, and replace *P_a_* by *P_c_* to optimize the training samples of RBFNN. MNNC can cluster datasets with different densities and shapes without specifying the number of clusters or scanning radius in advance, as shown in Figure 1c. Each cluster is displayed in a different color, and the cluster center is represented by blue circles. We construct the RBFNN based on MNNC. Each cluster corresponds to a hidden layer node, and the center of the cluster is the center of the node, as shown in Figure 1d. Thereafter, the RBFNN based on MNNC can be used to establish the relationship between the theoretical direction and reference direction of the magnetic microrobot’s locomotion. Therefore, if we obtain the theoretical driving direction of each step and input the theoretical direction into the RBFNN, the RBFNN outputs the reference direction. We can calculate the coil currents according to the reference direction, and accordingly, the microrobot moves along the theoretical direction driven by the magnetism generated by the coils. Finally, we can realize path tracking of the magnetic microrobot through the proposed method shown in Figure 1e. 

## 3. Clustering Algorithm Based on Nearest Neighbor

### 3.1. Typical Clustering Algorithm

A clustering algorithm is a typical unsupervised learning algorithm that is mainly used to automatically classify similar samples into a specific category. The main clustering methods can be divided into five methods: the partitioning method, hierarchical method, grid-based method, model-based method, and density-based method [24]. The partition method decomposes the data into n clusters, such that the items in each cluster are closely related to each other, for example, K-means algorithm. The calculation procedure in this algorithm is simple, but it is necessary to know the number of clusters of data in advance [25].

Balanced iterative reducing and clustering using hierarchies (BIRCH) is a typical representative of the hierarchical method that decomposes a given dataset hierarchically until a certain condition is met. Specifically, it can be categorized into “bottom-up” and “top-down” schemes [26]. This algorithm is not particularly suitable for non-convex datasets, and owing to the limit on the number of each node, the clustering result may deviate from the actual classification.

Clustering in QUEst (CLIQUE) is a clustering algorithm based on the grid method. In this algorithm, the data space is first divided into a grid structure of finite units, and all processing is based on a single unit. This algorithm is highly sensitive to parameters and cannot handle irregularly distributed data [27]. There is no iteration required in this method, but it is difficult to determine the density threshold, an important parameter in the algorithm. Model-based methods set a model for each cluster, and subsequently detect a dataset that satisfies this model adequately. Such a model may be the density distribution function of data points in space or other. The efficiency of this algorithm also needs to be improved [28]. The model-based method incorporates the probability and statistics approach and the neural network approach.

Density-based methods attempt to determine the high-density clusters separated by sparse regions. The size and shape of these clusters may be different. The most commonly used clustering algorithm based on density is DBSCAN. Although this algorithm does not necessitate knowledge of the number of classes the data is divided into in advance, knowledge of the radius and the minimum number of points is required [29].

### 3.2. Modified Nearest Neighbor-Based Clustering Algorithm for Training RBFNN

**Figure 2:**
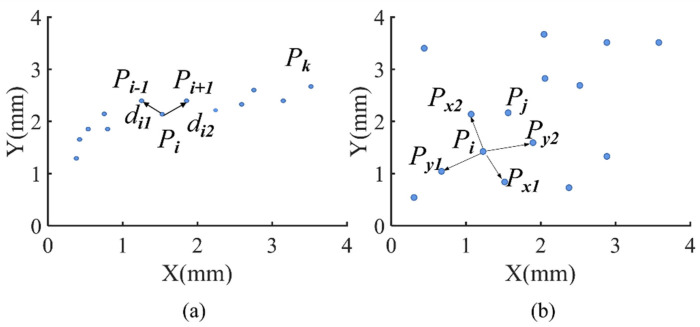
Sample distribution: (**a**) the training samples are dispersed with spatial sequence; (**b**) the training samples are dispersed without spatial sequence.

When we train the RBFNN for curve fitting and path tracking to obtain the structure parameters, clustering the training data to determine the center and number of basis functions of the hidden layer is an effective approach. The dataset in this case features obvious time or space characteristics. Here, we propose a simple clustering algorithm MNNC that clusters the samples according to the distance (*d_i_*) between the sample and its nearest neighbor, as shown in Figure 2 and Definition 1. Adjacent samples with similar d_i_ are categorized into the same cluster, and the method of searching for the nearest neighbor is modified. Only the distance between the sample and the preceding and the following samples needs to be calculated according to the property of the RBFNN training dataset. Thus, the calculation is significantly less than the other clustering algorithms, and only a single MNNC parameter requires to be determined.

**Figure 3:**
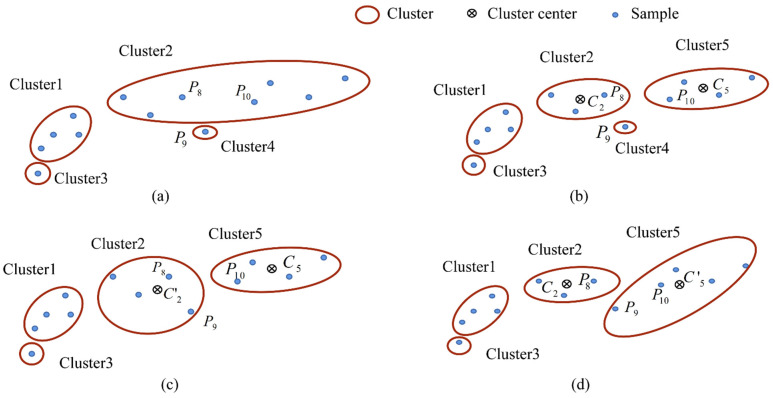
Clustering process of MNNC: (**a**) clustering result of training samples; (**b**) division of the cluster with discontinuous sample label into independent clusters; (**c**) Cluster 4 is merged into Cluster 2; (**d**) Cluster 4 is merged into Cluster 5.

The basic principle of the clustering algorithm is that similar samples are placed in the same cluster, where the similarity of two samples is described by the Euclidean distance of the two samples. Since the sample has the characteristics of time series, the nearest samples to sample $P_{i}$ are the adjacent samples $P_{i-1}$ and $P_{i+1}$, as shown in Figure 2a. The distances between the samples are $d_{i1}$ and $d_{i2}$, respectively, thus the nearest distance, $d_{i}$, of $P_{i}$ is the smaller of [$d_{i1}$,$d_{i2}$]. The calculation of this method is simpler than that of the other clustering algorithms. Thereafter, $d_{i}$ is divided into different levels, and the samples with the same $d_{i}$ level belong to the same cluster (Figure 3a). 

If the samples in one cluster are not adjacent, as shown in Figure 3a (where $P_{8}$ and $P_{10}$ are not adjacent samples), the cluster is divided at the breakpoint (Figure 3b). Finally, the small clusters are merged with the adjacent clusters, as shown in Figure 3c,d. The merging criterion is that the total distance change (ΔD) between all samples and the centroid should be the least, such that the adjacent samples with the similar distance characteristic form a cluster. The related definitions are executed in Algorithm 1 for the distance (*d_i_*), distance step (*d_step_*), and distance changes (ΔD).
**Algorithm 1.** MNNC.Input: training samples (*P*_1_,*P*_2_,……*P_k_*), minimum samples number (Nmin) of cluster.Output: clustering result1. **for** each sample *P_i_* do2.  Calculate the *d_i_* of *P_i_*; // referring to **Definition 1**.3. **end** for4. **for** each sample *P_i_* do // arrange the samples into clusters referring to the distance.5.  **if**
*d_i_* < *d_min_* + *d_step_* then *P_i_* ∈cluster1; // **Definition 2**6.  else if *d_i_* < *d_min_* + 2 × *d_step_* then *P_i_* ∈cluster2; 7.  else if *d_i_* < *d_min_* + 3 × *d_step_* then *P_i_* ∈cluster3;8.  else *P_i_* ∈cluster4;9. **end if**; end for10. for cluster(i) do // The clusters with discontinuous sample numbers are divided into two clusters at the discontinuity.11.  if the samples label of cluster(i) is not continuous then12.   Divide the cluster(i) into cluster(i1) and cluster(i2) whose samples label is continuous.13. **end if**; end for14. for cluster(i) do // merge the small cluster into the adjacent clusters referring to **Definition 3**.15.  **if** samples number of cluster(i) < Nmin **then**16.  **if** Δ*D*_*i*−1_ < Δ*D*_*i*+1_ then cluster(i − 1) = cluster(i − 1) + cluster(i);17.  else cluster(I + 1) = cluster(I + 1) + cluster(i);18. **end if**; end for19. **Return** clusters

**Definition** **1.** *The distance* *d_i_* *attribute of the sample,* $\left(P_{1},P_{2},\ldots \ldots ,P_{k}\right)$ *is the dataset of path tracking control system that includes* k *samples, and* $P{\left[x_{1},x_{2}\cdot \cdot \cdot \cdot \cdot \cdot x_{m}\right]}^{T}$ *is a single sample of the dataset that consists of* m *dimension components. The distance between* $P_{i}$ *and* $P_{i-1}$ *is* $d_{i1}$ *that can be expressed as [17]*

(1) $$d_{i1}=\sqrt{{\left(x_{i1}-x_{i-11}\right)}^{2}+{\left(x_{i2}-x_{i-12}\right)}^{2}+\ldots \ldots +{\left(x_{im}-x_{i-1m}\right)}^{2}}$$

*The distance between* $P_{i}$ *and* $P_{i+1}$ *is* $d_{i2}$ *that can be expressed as*

(2) $$d_{i2}=\sqrt{{\left(x_{i1}-x_{i+11}\right)}^{2}+{\left(x_{i2}-x_{i+12}\right)}^{2}+\ldots \ldots +{\left(x_{im}-x_{i+1m}\right)}^{2}}$$

*The minimal distance* $d_{i}$ *among* $\left(d_{i1},d_{i2}\right)$ *is expressed as*

(3) $$d_{i}=\min(d_{i1},d_{i2})$$

**Definition** **2.** *Distance step* *(d_step_)* $\left(d_{1},d_{2},\ldots \ldots ,d_{k}\right)$ *is the distance of the sample* $\left(P_{1},P_{2},\ldots \ldots ,P_{k}\right)$ *.*

(4) $$d_{\max}=\max(d_{1},d_{2},\ldots \ldots ,d_{k})$$

(5) $$d_{\min}=\min(d_{1},d_{2},\ldots \ldots ,d_{k})$$

*The distance step is calculated as follows, where* *H* *is the number of* *d_i_* *level determined by the user.*

(6) $$d_{step}=\frac{d_{\max}-d_{\min}}{H}$$

**Definition** **3.** *Distance change (* ΔD *)*

*The total distance of Cluster 2 and Cluster 5 (Figure 3b) is calculated before the merge operation.*

(7) $$D_{2}={\sum_{i=1}^{Quan_{2}}\left\|P_{i}-C_{2}\right\|}^{2}$$

(8) $$D_{5}={\sum_{i=1}^{Quan_{5}}\left\|P_{i}-C_{5}\right\|}^{2}$$

*where* $Quan_{2}$ *and* $Quan_{5}$ *are the quantity of samples in Cluster 2 and Cluster 5, respectively, and* $C_{2}$ *and* $C_{5}$ *are the centers of Cluster 2 and Cluster 5 as shown in Figure 3b, respectively; the center of Cluster 2 can be determined using Equation (9), and we can obtain the centers of the other clusters similarly.*

(9) $$C_{2m}=\frac{x_{1m}+x_{2m}+\ldots \ldots +x_{Um}}{U}$$

*where* $C_{2m}$ *is the component* m *of the center of Cluster 2, and* $x_{1m}$ *is the component* m *of sample 1 of Cluster 2. The total distances of Cluster 2 and Cluster 5 are calculated after the merge operation. If Cluster 4 is merged into Cluster 2, the center of Cluster 2 becomes C_2′_, as shown in Figure 3c.*

(10) $$D{’}_{2}={\sum_{i=1}^{U’}\left\|P_{i}-C{’}_{2}\right\|}^{2}$$

*If Cluster 4 is merged into Cluster 5, the center of Cluster 5 becomes C_5′_, as shown in Figure 3d.*

(11) $$D{’}_{5}={\sum_{i=1}^{V’}\left\|P_{i}-C{’}_{5}\right\|}^{2}$$

*Therefore, the distance change (* ΔD *) is*

(12) $$\Delta D_{2}=\left|D{’}_{2}-D_{2}\right|$$

(13) $$\Delta D_{5}=\left|D{’}_{5}-D_{5}\right|$$

**Figure 4:**
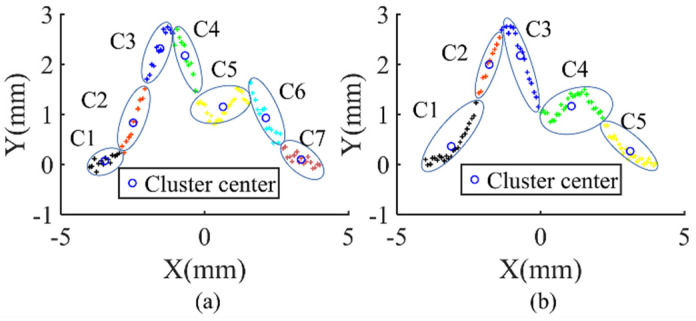
Clustering results: (**a**) the training samples are divided into seven clusters; (**b**) the training samples are divided into five clusters.

Figure 4 shows the results of Algorithm 1 for clustering. To test the algorithm, we selected 101 points from the curve $y=1.1(1-x+2x^{2})e^{-x^{2}/2}$ and combined them with random noise. We set the number of distance level *H* = 4, and tests were conducted twice with different datasets. Under these conditions, Algorithm 1 automatically generated seven and five clusters according to the distance properties (*d_i_*, *d_step_, and* ΔD), implying that MNNC is adaptive to the different density and can tune the number of clusters automatically. Merging clusters with a small number of samples (the number is not limited to 1) into other clusters can reduce the number of clusters. In this manner, when the clustering algorithm is applied along with other intelligent algorithms, the speed of the intelligent algorithm can be improved.

### 3.3. Enhancement of MNNC Performance

The clustering algorithm can use the samples with a time sequence or spatial sequence, as shown in Figure 2a; the nearest neighbor of sample *P_i_* is either *P_i_*_−1_ or *P_i_*_+1_. This clustering algorithm is suitable for curve fitting and path tracking control. Furthermore, the samples are randomly distributed without the time and spatial sequences, as shown in Figure 2b. In this case, the nearest neighbor of *P_i_* may be in any direction, and we need to calculate the distances from *P_i_* to its neighbors for determining *d_i_*. The detailed calculation is shown in Definition 4. After we obtain the distances $\left(d_{1},d_{2},\ldots \ldots ,d_{k}\right)$, that is, the $d_{i}$ of the samples, we set $d_{\max}=\min(d_{1},d_{2},\ldots \ldots ,d_{k})$. Thereafter, the sample *P_m_* is determined, whose distance to *P_i_* is less than $d_{\max}$. These samples and *P_i_* form a neighbor cluster of *P_i_*. Similarly, the neighbor clusters of the other samples can be established. If the sample *P_max_* owns the distance attribute of *d_max_*, and its neighbor cluster contains only two samples, we define *P_max_* as an abnormal sample and delete this sample. Next, we set the updated maximum distance, and subsequently establish the neighbor cluster of each sample again. If there are several neighbor clusters containing the same samples, then these neighbor clusters merge into one cluster. The execution process proceeds based on Algorithm 2.

**Definition 4.***Distance attribute of the sample (*$d_{i}$*): To reduce the calculation, the samples are sorted byxandy, respectively.P_x_*_1_*andP_x_*_2_*are the preceding and following samples relative to sampleP_i_, sorted by x.P_y_*_1_*andP_y_*_2_*are the preceding and following samples relative to sampleP_i_sorted byy*. *d_x_*_1_, *d_x_*_2_, *d_y_*_1_*, andd_γ_*_2_*are the distances betweenP_i_andP_x_*_1_, *P_x_*_2_, *P_y_*_1,_*andP_y_*_2_*, respectively.*

(14) $$d_{x1}=\sqrt{{\left(x_{x1}-x_{i}\right)}^{2}+{\left(y_{x1}-y_{i}\right)}^{2}}$$

(15) $$d_{x2}=\sqrt{{\left(x_{x2}-x_{i}\right)}^{2}+{\left(y_{x2}-y_{i}\right)}^{2}}$$

(16) $$d_{y1}=\sqrt{{\left(x_{y1}-x_{i}\right)}^{2}+{\left(y_{y1}-y_{i}\right)}^{2}}$$

(17) $$d_{y2}=\sqrt{{\left(x_{y2}-x_{i}\right)}^{2}+{\left(y_{y2}-y_{i}\right)}^{2}}$$

*d_i_*_0_ *can be expressed as*

(18) $$d_{i0}=\min(d_{x1},d_{x2},d_{y1},d_{y2})$$

*The sampleP_j_*; *x_j_* and *y_j_ of* *P_j_* *satisfy*

(19) $$\left|x_{j}-x_{i}\right|\leq d_{i0}$$

(20) $$\left|y_{j}-y_{i}\right|\leq d_{i0}$$

*The distance between* *P_j_ and* *P_i_*_:_ *can be calculated using*

(21) $$d_{j}=\sqrt{{\left(x_{j}-x_{i}\right)}^{2}+{\left(y_{j}-y_{i}\right)}^{2}}$$

*Finally, the distance attribute of P_i_ is*

(22) $$d_{i}=\min(d_{1},d_{2},\ldots \ldots ,d_{j})$$

**Algorithm 2.** MNNC for the path tracking of magnetic microrobot.Input: training samples (*P*_1_,*P*_2_,……*P_k_*).Output: clustering result.1. **for** each sample *P_i_* do2.  Calculate the *d_i_* of *P_i_*; // referring to **Definition 4**.3. **end** for4. *d_max_* = max(*d*_1_,*d*_2_,……*d_k_*); *d_min_* = min(*d*_1_,*d*_2_,……*d_k_*);5. *d_step_* = (*d_max_* + *d_min_*)/H; //H is distance level number that is determined by user.6. **for** each distance level;7. **for** each sample *P_i_* do // find the nearest neighbors of *P_i_* and establish the neighbor clusers.8.  Find the smaple *P_m_* which ‖*P_m_*−*P_i_*‖ ≤ *d_min_* + H × *d_step_*;9.  Construct cluster(i) = (*P_i_*, *P_m_*);10. **end** for11. **for** each cluster(i) do // if clusters contain same sample, then merge these clusters into one cluster.12.  **If** cluster(i) ∩ cluster(j) ≠ Ø then cluster(i) = cluster(i) + cluster(j);13. **end if**; end for14. **end** for15. merge the small cluster into nearest cluster16. **Return** clusters

**Figure 5:**
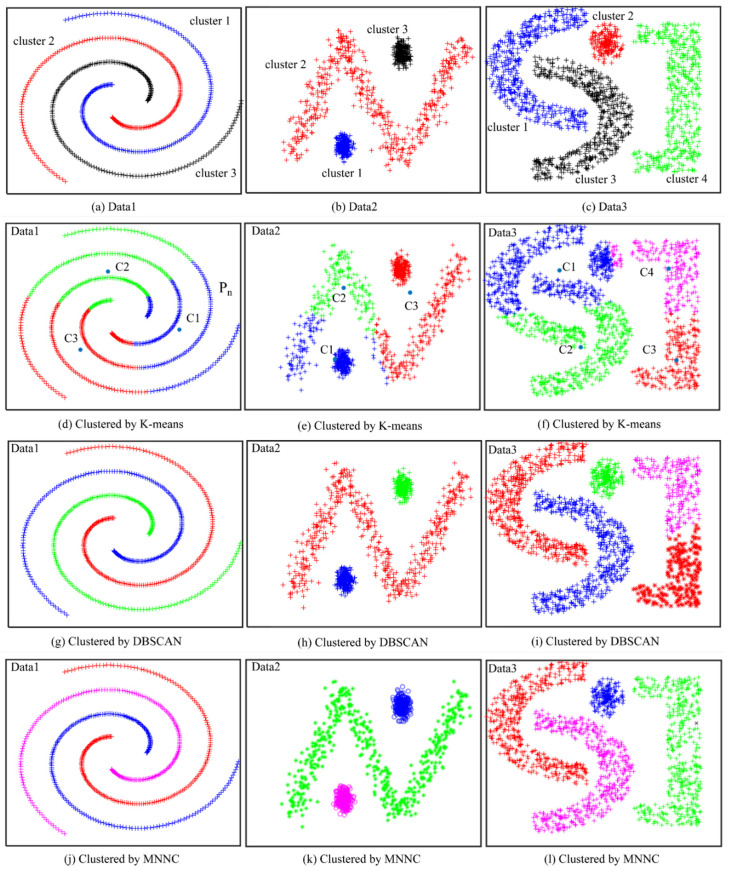
Clustering results of different clustering algorithms: (**a**–**c**) are the reference results; (**d**–**f**) are clustered by k-means; (**g**–**i**) are clustered by DBSCAN; (**j**–**l**) are clustered by MNNC.

It can be observed from the previous steps that MNNC for path tracking does not require us to pre-select important parameters based on experience. Because there is no iterative process, the calculation is not large in the algorithm. Furthermore, this clustering algorithm is also suitable for multi-dimensional samples. To verify the effectiveness of the clustering algorithm, we used MNNC, K-means, and DBSCAN to perform clustering analysis on the same samples, as shown in Figure 5.

We generated three synthetic datasets called Data 1 (Figure 5a), Data 2 (Figure 5b), and Data 3 (Figure 5c) containing 600, 1001, and 1650 samples, respectively. Data 1 consists of three clusters that are marked with ‘blue +’, ‘red +’, and ‘black +’, respectively. Data 2 also consists of three clusters that are marked with ‘blue +’, ‘red +’, and ‘black +’, respectively. Data3 consists of four clusters that are marked with ‘blue +’, ‘red +’, ‘black +’, and ‘green +’, respectively.

Figure 5d–f are the clustering results of Data 1, Data 2, and Data 3 obtained by k-means, respectively. Prior to the cluster analysis of Data 1, Data 2, and Data 3 by k-means, we set the parameter *K* to 3, 3, and 4, respectively, but the clustering results still remain incorrect. 

Data 1 was divided into three clusters that were marked with ‘blue +’, ‘green +’, and ‘red +’, as shown in Figure 5d. As the reference result in Figure 5a shows, the samples of every cluster form a spiral. However, the cluster formations were changed when Data 1 was clustered by k-means. The changed cluster formed around each center of the clusters (C1, C2, and C3); blue cluster is the area around C1, green cluster is the area around C2, the red cluster is the area around C3. This is because of the principle of k-means that clusters the dataset based on the distance between samples and cluster centers. For example, we assume that C1, C2, and C3 are the centers of blue, green, and red clusters, respectively. *P_n_* is any sample of the blue cluster, as shown in Figure 5d. For the sample *P_n_*, C1 is the nearest cluster center among C1, C2, and C3. Therefore, sample *P_n_* becomes one of the samples of the blue cluster. Similar results are shown in Figure 5e. The cluster marked with ‘red +’ consists of some samples indicated by circles and some samples indicated by “N” because C3 is the nearest cluster center for these samples. The k-means algorithm divides the samples in “N” into three different clusters that are marked with ‘blue +’, ‘green +’, and ‘red +’, respectively. However, in the reference result (Figure 5b), the samples in “N” form a single cluster (red +), and the samples in one circle form a single cluster as well. In Figure 5f, Data 3 is divided into four clusters by k-means algorithm, but the green cluster contains samples into two arcs because C2 becomes the nearest cluster center for the samples in this case. The blue cluster includes the samples of a circle and two arcs, and the samples in “I” are divided into two clusters. However, the reference result (Figure 5b) shows that the samples in one arc should form a single cluster, and the samples in circle and “I” should also form a single cluster, respectively. According to Figure 5d–f we can conclude that k-means was unsuccessful in clustering Data 1, Data 2, and Data 3. 

During the simulation, we set *R* (scanning radius) of DBSCAN equal to the maximum distance (*d_max_*) of MNNC, and set the same *M_p_* (minimal points number) for Data 1, Data 2, and Data 3. The clustering results of Data 1, Data 2, and Data 3 by DBSCAN are shown in Figure 5g–i respectively. As shown in Figure 5g, DBSCAN divided Data 1 into three clusters precisely, as shown in Figure 5g; it placed the samples of one spiral in an independent cluster. DBSCAN generated three clusters for Data 2, as shown in Figure 5h; the three clusters were marked with ‘blue +’, ‘green +’, and ‘red +’, respectively. However, as shown in Figure 5i DBSCAN divided the samples in “I” into two clusters that were marked with ‘pink +’, and ‘black +’. The clustering result of Data 3 by DBSCAN is not equivalent to the reference result for Data 3 that shows that the samples in “I” belong to a single cluster. This is because DBSCAN not only depends on the parameter *R*, but also on the parameter *M_p_*. However, at this time, *M_p_* is not suitable for Data 3 anymore, implying that we should define two correct parameters of DBSCAN for different cases based on experience.

Figure 5j–l are the clustering results of Data 1, Data 2, and Data 3 usingMNNC, respectively. Figure 5j,k show that MNNC generated equivalent clusters for Data 1 and Data 2. MNNC divided the samples of Data 1 and Data 2 into three clusters that were marked with ‘blue +’, ‘green +’, and ‘red +’, respectively. MNNC almost clustered Data 3 correctly, except it regarded one sample in “I” as an abnormal sample (marked with “black ×”), as shown in Figure 5l. This is because the sample, marked with “black ×”, features the largest *d_i_*, and there are no similar samples around it. 

Therefore, we can conclude that MNNC features the best clustering function for these datasets. Simultaneously, MNNC does not require users to decide the parameters, whereas both DBSCAN and k-mean require users to define two parameters. To clearly describe the clustering results, we used ACC and ARI to evaluate the clustering results [30]. The results are shown in Table 1. 

As shown in Table 1, the ACC and ARI of three datasets of k-means clustering are much smaller than those of DBSCAN and MNNC, implying that the clustering results by k-means are lower than those obtained by DBSCAN and MNNC. The ACC and ARI of the spiral and zigzag of DBSCAN and MNNC are all 1, indicating that both DBSCAN and MNNC cluster those two datasets precisely. The ACC index of C4 of DBSCAN and MNNC are 0.8558 and 0.9994, respectively. The ARI index of C4 by DBSCAN and MNNC are 0.9019 and 0.9994, respectively. Both indexes of DBSCAN are smaller than those of MNNC which implies that the clustering results of MNNC are better than those of DBSCAN. The result of clustering index is similar to the clustering result, as shown in Figure 5.

## 4. Adjustment of Training Samples Based on MNNC

**Figure 6:**
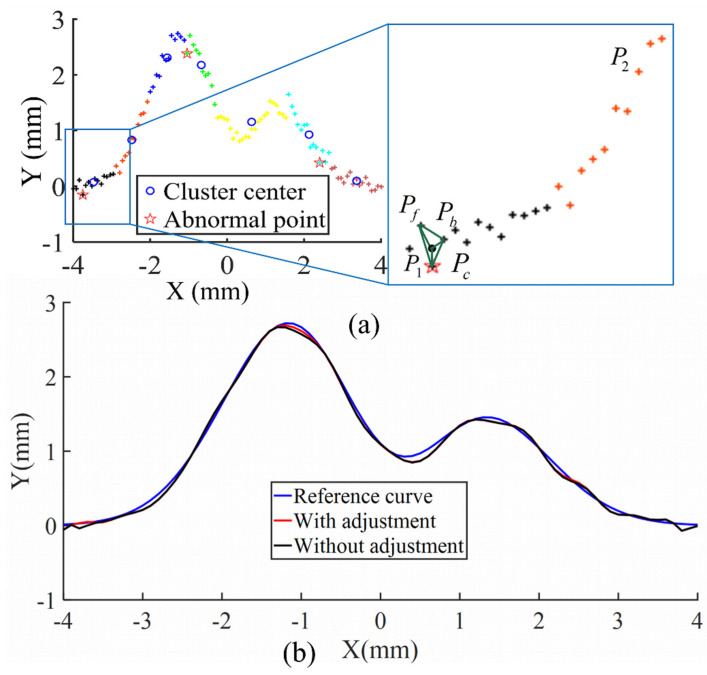
(**a**) Scheme of analysis of abnormal sample; (**b**) curve fitting results.

The abnormal samples can be optimized by unsupervised methods before the intelligent algorithm parameters are defined. Therefore, the unsupervised methods can significantly improve the predictive ability of intelligent algorithm models [31]. Abnormal training samples always affect the operating efficiency of neural networks [32]; it is necessary to detect the abnormal samples and treat them. Training samples for the path tracking system with abnormal samples are shown in Figure 6a. The abnormal samples are marked as stars that reduce the learning effect of the algorithm for path tacking such as RBFNN. We can detect the abnormal samples by MNNC that requires fewer parameters than the other algorithms. After defining the abnormal samples, we can delete them directly, but it is not particularly effective for curve fitting or path tracking control. 

Particularly, when the number of training samples is not large, insufficient training samples also reduce the accuracy of RBFNN. It is effective to adjust the abnormal samples to normal samples. The process of detecting and adjusting abnormal samples is performed by MNNC. First, the training samples are clustered by MNNC. There are some independent samples because they are far away from the neighbors, such as *P*_1_ and *P*_2_ that are the abnormal and normal samples, respectively. We should distinguish between *P*_1_ and *P*_2_. Therefore, a triangle is formed by the samples of $P_{1}$, $P_{f}$, and $P_{b}$. The samples $P_{f}$ and $P_{b}$ are neighbors of $P_{1}$, as shown in Figure 6a. Thereafter, we calculate the distance from the center of the triangle ($P_{c}$) to *P*_1_, $P_{f}$, and $P_{b}$, respectively. On one hand, if the distance *(d_c__*_1_) between $P_{c}$ and *P*_1_ is not larger than that of $P_{f}$ and $P_{b}$, we define *P*_1_ as a normal sample. On the other hand, when the *d*_*c*_1_ is larger than that of $P_{f}$ and $P_{b}$, the sample *P*_1_ becomes the abnormal sample and moves to $P_{c}$.

We tested the effect of training sample adjustment. The training samples were obtained from different curves that were combined with random noise or some specific noise. The results are shown in Figure 6b and summarized in Table 2. From Figure 6b and Table 2, we can observe that the fitting errors of the 2D curve mixed with random noise are 3.0793 when the abnormal samples are not adjusted; but the fitting errors of the same dataset are only 2.8145 after the abnormal samples are adjusted. Furthermore, the fitting errors of the 2D curve mixed with six noise points without and with abnormal sample adjustment are 2.7281 and 1.3292, respectively. The modified effect of the 3D curve is not comparable to that of the 2D curve; the fitting errors decrease from 516.6542 to 485.3374. Therefore, we can conclude that the adjustment of abnormal samples can improve the curve fitting accuracy. Particularly, the accuracy is improved by approximately 50% when there are only six abnormal samples. Because these six abnormal samples deviate far from the normal samples, these six samples change considerably after they are adjusted to normal samples. Therefore, the accuracy of the entire curve fitting is significantly improved. All the simulations were performed in Matlab.

## 5. Application of RBFNN in Path Tracking for a Spiral-Type Magnetic Microrobot

**Figure 7:**
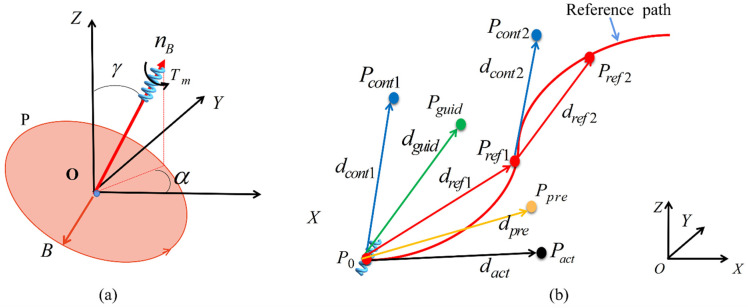
(**a**) Magnetic field vector; (**b**) scheme of path tracking: the green arrow is the guidance direction, the red arrow is the desired direction, the yellow arrow is the predicted direction, the blue arrow is the control direction, and the black arrow is the actual directions.

Figure 7a shows the control method of a spiral-type magnetic microrobot using rotating magnetic field (RMF) control. The robot is synchronized by the applied RMF and driven by magnetic torque. A rotation of the robot generates propulsive force via the screw mechanism. The driving magnetic torque $T_{m}$ can be expressed as follows [33]:

(23) $$T_{m}=VM\times B$$

where *V* is the volume of microrobot, *M* is the magnetization, and *B* is the external magnetic flux density. The magnetized direction of the robot is the radial direction. The external magnetic field is a uniform RMF and is generated by a three-axis Helmholtz coil. We assume that a magnetic field *B* generated by 3D Helmholtz coils rotates in plane P. Thus, the normal vector (*^n^B*) of plane P represents the movement direction of the robot. In addition, because the control angles of γ and α determine the position of plane P, the control of two angles determines the steering of the robot. The normal vector $n_{B}$ and magnetic field B can be described as follows:

(24) $$n_{B}={\left[\sin(\gamma )\cos(\alpha ),\sin(\gamma )\sin(\alpha ),\cos(\gamma )\right]}^{T}$$

(25) $$B=\left[\begin{array}{c} B_{x}\\ B_{y}\\ B_{z} \end{array}\right]=B_{0}\left[\begin{array}{c} \cos(\gamma )\cos(\alpha )\sin(\omega t)+\sin(\alpha )\cos(\omega t)\\ \cos(\gamma )\sin(\alpha )\sin(\omega t)-\cos(\alpha )\cos(\omega t)\\ -\sin(\gamma )\sin(\omega t) \end{array}\right]$$

where $B_{0}$ is the norm of B; γ is the polar angle, and α is the azimuthal angle.

We assume that there are control errors resulting from various environmental factors. When we plan to drive the robot from the present position $P_{o}$ to the reference target position $P_{ref1}$ along the reference direction $d_{ref1}$, the robot may arrive at the actual position $P_{act}$ because of locomotion error between the actual and reference positions. Therefore, when we drive the robot to move along the control direction $d_{act1}$ to compensate for the locomotion error, the robot may reach the position $P_{ref1}$, as shown in Figure 7b. If there is no error between the actual and reference positions, the control direction *d_c_*_1_ is matched to the reference direction $d_{ref1}$ by training RBFNN. Next, when the robot arrives the position $P_{ref1}$, we can obtain the next reference target position $P_{ref2}$ and the reference direction $d_{ref2}$. Because the locomotion error is different in each locomotion step, we can obtain the corresponding compensation by driving the robot along the control direction. Therefore, driving the robot to move along the control direction *d_ci_*, the robot can reach each reference position *P_r_* along the reference path. To decide the steering direction of the robot along the reference direction, the two angles of $\gamma_{ref}$ and $\alpha_{ref}$ are input to the RBFNN, and we obtain the actual control angles of $\gamma_{cont}$ and $\alpha_{cont}$ by RBFNN for the controlling plane of RMF. To achieve this aim, it is necessary to develop a locomotion control system for the robot that is a nonlinear system. For nonlinear locomotion control systems, some researchers use RBFNN to simulate dynamic models [34,35]. However, the large number of parameters of these methods make the control system highly complicated. The neural network controller is a nonlinear mapping system; it has been proved that any smooth function can be represented by a three-layer neural network with sufficient hidden neurons [36]. Finally, RBFNN can be used to establish the relationship between the reference ($\gamma_{ref}$ and $\alpha_{ref}$) and control angles ($\gamma_{cont}$ and $\alpha_{cont}$) after it is trained. 

**Figure 8:**
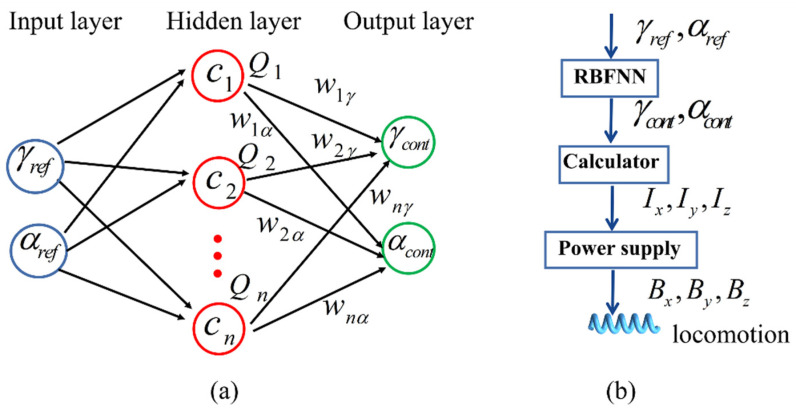
Scheme of RBFNN and control system: (**a**) structure of RBFNN for path tracking; (**b**) flow chart of control system.

Generally, RBFNN includes three layers: input, hidden, and output layers, as shown in Figure 8a. In this study, we used MNNC to train the RBFNN to develop its structure. 

The input layer of RBFNN includes two neurons: reference angle $\gamma_{ref}$ and $\alpha_{ref}$. The number of neuron and center of hidden layer are determined by MNNC after the RBFNN is trained. Accordingly, the output of the hidden layer can be obtained as

(26) $$Q_{n}=e^{\left(-\frac{{\left\|(\gamma_{ref},\alpha_{ref})-c_{n}\right\|}^{2}}{2\sigma^{2}}\right)}$$

where *c_n_* is the center of hidden layer neurons that is decided by the MNNC. σ is the width of basis function that can be expressed as [37].

(27) $$\sigma =\frac{d_{c}}{\sqrt{2n}}$$

where *d_c_* is the maximum distance among the neuron centers of hidden layer; *n* is the quantity of neuron units of the hidden layer, and both of them can be obtained by MNNC.

The output layer includes two neurons: control angles of $\gamma_{cont}$ and $\alpha_{cont}$. They can be obtained by RBFNN according to

(28) $$\{\begin{array}{c} \gamma_{cont}={\sum_{i=1}^{n}Q_{i}w}_{i\gamma }\\ \alpha_{cont}={\sum_{i=1}^{n}Q_{i}w}_{i\alpha } \end{array}$$

where $w_{i\gamma }$ and $w_{i\alpha }$ is the weight of $\gamma_{cont}$ and $\alpha_{cont}$, respectively, that can be obtained by training the RBFNN based on the training samples.

Thus, when we input the reference angle into RBFNN, we can obtain the control angle from the output layer. Next, we calculate the driving current to generate RMF, as shown in Figure 8b. Using Equations (24) and (25), we obtain the driving magnetic field B that is the uniform magnetic field generated in the Helmholtz coils; the relationship between the magnetic field and coil current can be expressed as follows [33]:

(29) $$B=\mu_{0}NK_{B}I$$

where $\mu_{0}$ is the permeability of vacuum, N is number of turns of coil, $K_{B}$ is the magnetic field coefficient of Helmholtz coil, and I is the coil current. 

**Figure 9:**
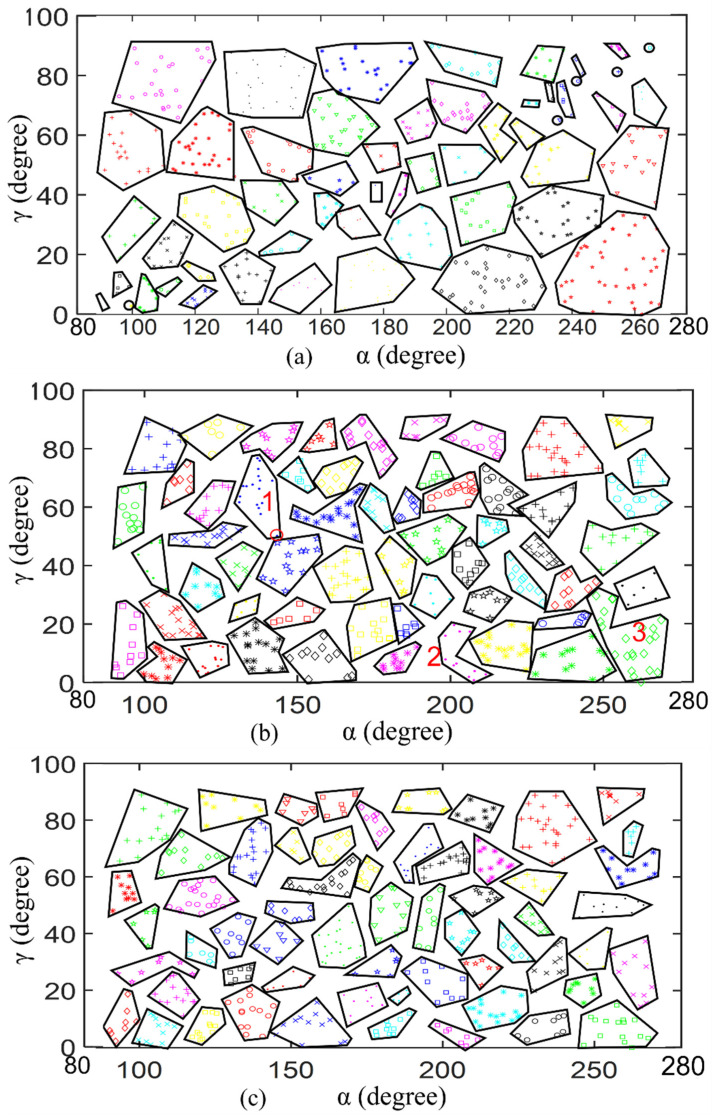
Clustering results of training data: (**a**) clustered by K-means;.(**b**) clustered by DBSCAN; (**c**) clustered by MNNC.

To verify the ability of the proposed method for path tracking, we performed simulation using RBFNN with MNNC for path tracking. We generated 600 training samples to train the RBFNN to compare the performance of the clustering using MNNC, DBSCAN, and k-means applied to the path tracking simulation, as shown in Figure 9. The 600 samples were composed of 59 clusters for comparison under the same conditions. Thus, we could determine the neuron number and center of the hidden layer of the RBFNN, and obtain the width of each basis function of the RBFNN hidden layer.

Although the suitable k and accuracy of k-means are set, there are still some problems in the clustering result obtained by k-means algorithm. For example, there are many clusters (marked with circles) included for only one sample, as shown in Figure 9a. These clusters are closely spaced and could be merged into a large cluster. Although we adjusted the scanning radius and minimal sample number of DBSCAN for a long time, the clustering result was still not satisfactory. For example, there is a sample (marked with a red circle) far away from another sample in Cluster 1 that should be categorized as a neighboring cluster, as shown in Figure 9b. There are also samples far away from the other samples in Cluster 2 and Cluster 3, as shown in Figure 9b. Nonetheless, the similar data were placed in the same cluster by MNNC, as shown in Figure 9c.

As described above, after the training data were clustered, the neuron number was set as the cluster number of training data, and the cluster center was set as the neuron center of the hidden layer. We calculated and adjusted the width of basis function and weights between the hidden layer neurons and output layer neurons while training the RBFNN. Hence, the relationship between reference direction and control direction are established by RBFNN.

After the relationship between the control and reference angles are established, if we place any reference angle into RBFNN, we can obtain the corresponding control angle. To obtain the reference angle, we should obtain the reference target position first. We selected 30 points as the target points $P_{t}(x_{t},y_{t},z_{t})$ in the reference path of the robot, and the path equation can be expressed as follows:

(30) $$\{\begin{array}{c} x=4\cos(t)\\ y=4\sin(t)\\ z=3t/\pi \end{array}$$

(31) $$\gamma_{ref}=arc\cos\left(\frac{z_{t}-z_{i}}{\sqrt{{\left(x_{t}-x_{i}\right)}^{2}+{\left(y_{t}-y_{i}\right)}^{2}+{\left(z_{t}-z_{i}\right)}^{2}}}\right)$$

(32) $$\alpha_{ref}=arc\cos\left(\frac{x_{t}-x_{i}}{\sqrt{{\left(x_{t}-x_{i}\right)}^{2}+{\left(y_{t}-y_{i}\right)}^{2}}}\right)$$

These reference angles were mixed with the input data of training samples, and subsequently, they were combined with the compensation angle ($\alpha_{comp}$,$\gamma_{comp}$) to obtain the control directions $d_{cont}$ with the components of $\alpha_{cont}$ and $\gamma_{cont}$, as shown in Figure 7b. Upon inputting $\gamma_{ref}$ and $\alpha_{ref}$ to RBFNN, the control angles were obtained. Nonetheless, because of the fitting error of RBFNN, there was some deviation between the output data and control directions. The output of RBFNN at this time acts as the guidance direction $d_{guid}$ that includes the components of $\alpha_{guid}$ and $\gamma_{guid}$, as shown in Figure 7b. $\alpha_{guid}$ and $\gamma_{guid}$ can be obtained from RBFNN according to

(33) $$\gamma_{guid}={\sum_{i=1}^{n}e^{\left(-\frac{{\left\|(\alpha_{ref},\gamma_{ref})-c_{i}\right\|}^{2}}{2{\sigma_{i}}^{2}}\right)}w}_{i\gamma }$$

(34) $$\alpha_{guid}={\sum_{i=1}^{n}e^{\left(-\frac{{\left\|(\alpha_{ref},\gamma_{ref})-c_{i}\right\|}^{2}}{2{\sigma_{i}}^{2}}\right)}w}_{i\alpha }$$

where n, $c_{i}$, $\sigma_{i}$, $w_{i\gamma }$, and $w_{i\alpha }$ are the neuron number of the hidden layer, the neuron center of the hidden layer, the width of the basis function of the hidden layer, the weight of $\gamma_{guid}$, and the weight of $\alpha_{guid}$, respectively. The parameters can be obtained after training RBFNN. When the reference angles of $\gamma_{ref}$ and $\alpha_{ref}$ are input to RBFNN, the control angles $\gamma_{cont}$ and $\alpha_{cont}$ are obtained for the path tracking of the spiral-type magnetic microrobot. Comparing the guidance and control directions, the test errors of radial basis function neural network, representing its accuracy, can be obtained. We trained and tested the RBFNN based on k-means, DBSCAN, and MNNC, respectively. These tests were based on the same learning rate, iteration number, the momentum factor, training samples, and test samples. The iteration number, learning rate, and momentum factor of the training process are 5000, 0.09, and 0.03, respectively. The results are shown in Table 3.

As can be observed from Table 3, the cluster numbers of all clustering algorithms are 59, and the training parameters are the same, but the training and test errors are different. The training errors of radial basis function neural network based on k-means, DBSCAN, and MNNC are 2.27°, 2.22°, and 2.10°, respectively, and the test errors of the radial basis function neural networks based on k-means, DBSCAN, and MNNC are 2.89°, 2.24°, and 2.21°, respectively. Therefore, the radial basis function neural network based on MNNC provides the best test result. Accordingly, we can conclude that MNNC is the best algorithm for training the RBFNN for establishing the relationship between the control angle and the reference angle.

Based on the guidance direction angles, the coil current can be obtained from the calculator of the control system, as shown in Figure 8b. We selected seven positions of the test samples evenly, the coil current equations of which are shown in Table 4.

After current is input into the coils, the coils generate the magnetic field $B_{x}$, $B_{y}$, and $B_{z}$. These magnetic fields are combined into a rotating magnetic field that drive the spring-type robot to the predicted target $P_{pre}$ along the predicted direction $d_{pre}$, as shown in Figure 7b. Here, we calculate the predicted angles $\alpha_{pre}$ and $\gamma_{pre}$ based on

(35) $$\{\begin{array}{c} \alpha_{pre}=\alpha_{guid}-\alpha_{comp}\\ \gamma_{pre}=\gamma_{guid}-\gamma_{comp} \end{array}$$

where $\alpha_{comp}$ and $\gamma_{comp}$ are the compensation angles that are generated during the sample generation process.

We can obtain the angle error ratio of α and γ using

(36) $$\{\begin{array}{c} err_{alpha}=\frac{\alpha_{pre}-\alpha_{ref}}{\alpha_{ref}}\times 100\%\\ err_{gamma}=\frac{\gamma_{pre}-\gamma_{ref}}{\gamma_{ref}}\times 100\% \end{array}$$

The coordinate of the predicted target $P_{pre}$ can be obtained from

(37) $$\{\begin{array}{c} x_{pre}=\left\|P_{ref}-P_{0}\right\|\sin(\gamma_{pre})\cos(\alpha_{pre})\\ y_{pre}=\left\|P_{ref}-P_{0}\right\|\sin(\gamma_{pre})\sin(\alpha_{pre})\\ z_{pre}=\left\|P_{ref}-P_{0}\right\|\cos(\gamma_{pre}) \end{array}$$

Accordingly, the position error is calculated based on

(38) $$err_{position}=\frac{\left\|P_{pre}-P_{ref}\right\|}{\left\|P_{ref}-P_{0}\right\|}\times 100\%$$

**Figure 10:**
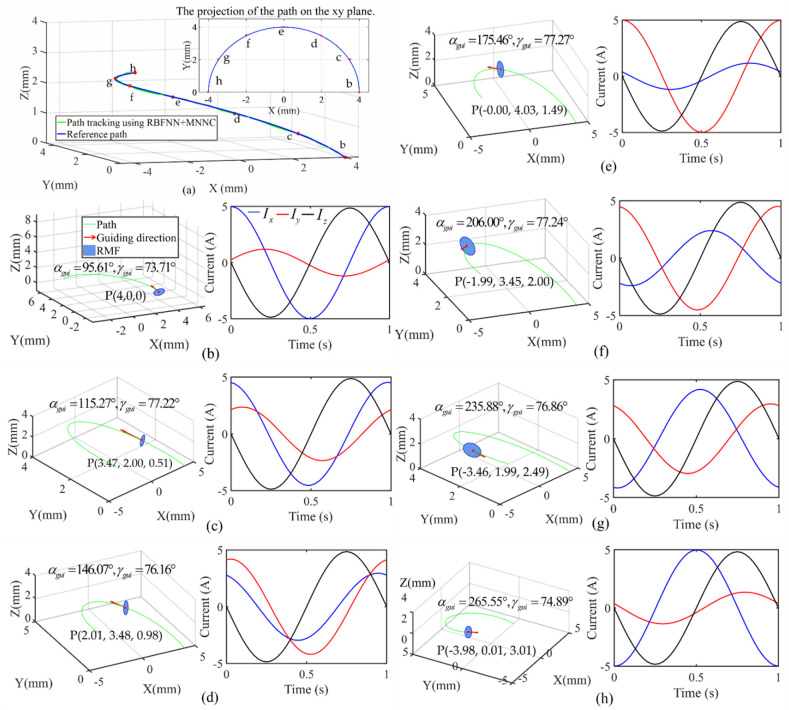
Path tracking of magnetic microrobot by RBFNN: (**a**) simulation results of path tracking by MNNC-based RBFNN; (**b**–**h**) show the rotating plan of the magnetic field and the movement direction at different positions of the path, and the coil current of *X*, *Y*, and *Z*-axis in one cycle; the rotating frequency of magnetic field is 1 Hz. For improved visualization of the parameters, the view angle for each figure is different.

The simulation result of seven positions are shown in Table 5 and Figure 10.

Because the control angles determine the steering direction, the two control angles automatically generate three current signals to produce an RMF and determine the position of the plane of RMF, as shown in Figure 10. Figure 10a shows the reference path and the simulation result of the path tracking on the reference path using RBFNN along with MNNC. Figure 10b–h shows the seven positions of the robot and their control conditions according to the changes in the control angles. There are the coordinates of position, reference path (green curve), plane of RMF (blue circle plan), movement direction (red arrow), or the direction of the normal vector of the plane of the rotating magnetic field, the control angles, and the generated currents in one cycle of the three-axis Helmholtz coils. At the seven positions on the path, the generated current signals for the RMFs are summarized in Table 4. We assumed that the frequency of RMF was 1 Hz and the coefficients $\mu_{0}NK_{B}$ of the coil were normalized as 1. In addition, the robot has a right-handed screw mechanism, and the rotating direction of the magnetic field is clockwise. In this case, the direction of normal vector becomes the movement direction of the robot, and the control angles become the steering direction of the robot.

When the starting position of the robot was at point (4,0,0), the driving angles ($\gamma_{ref}$ and $\alpha_{ref}$) and guidance angles ($\gamma_{guid}$ and $\alpha_{guid}$) were calculated as $\gamma_{ref}$ = 76.57°, $\alpha_{ref}$ = 93°, $\gamma_{guid}$ = 73.71°, and $\alpha_{guid}$ = 95.61°, respectively, as shown in Figure 10b. Under the conditions, the three generated currents are $I_{x}=0.999\sin(360t+90.69{}^{\circ})$, $I_{y}=0.238\sin(360t+12.72{}^{\circ})$, and $I_{z}=-0.973\sin(360t)$, respectively. When the guidance angles of $\gamma_{guid}$ and $\alpha_{guid}$ are 77.22° and 115.27°, the robot reaches Position c, as shown in Figure 10c. Moving from Position b to c, we can confirm that the current profiles are changed by the variation of the guidance angles (steering angles). $\alpha_{guid}$ allows the RMF plane to rotate around the *Z*-axis and the changes in the $\gamma_{guid}$ cause the RMF plane to rotate around *X*-axis or *Y*-axis or both (Figure 7a). Therefore, when there is no angular change in the moving path of the robot, the generated current profiles are constant, while the current profiles are changed when there is an angular change in the moving path. Through the phase difference and amplitude of the currents, the movement direction of the robot is determined. 

In Figure 10c, the present position of the microrobot is (3.47, 2.00, 0.51). The guidance angle $\gamma_{guid}$ and $\alpha_{guid}$ are 77.22° and 115.27°, respectively, that were obtained by RBFNN. The control system calculated the corresponding coils current along the *x*-axis, *y*-axis, and *z*-axis indicated by the blue, red, and green curves, respectively, as shown in Figure 10c. The amplitudes of $I_{x}$, $I_{y}$, and $I_{z}$ are 0.909, 0.471, and 0.975, respectively, as shown in Table 4. From Table 4, we can observe that the phase of $I_{x}$, $I_{y}$, and $I_{z}$ are 95.96°, 64.89°, and 0, respectively. Comparing Figure 10b,c the current in the *z*-axis coils of these two cases are similar because the angle $\gamma_{guid}$ changes negligibly. However, there are large changes in the curve corresponding to the current in the *x*-axis and *y*-axis coils because the angle $\alpha_{guid}$ changes significantly; therefore, we can obtain the results using Equations (24) and (25). The similar control process was implemented for the other positions, and the corresponding results are shown in Figure 10c–g and Table 5. The error ratios of path tracking are shown in Table 5. When the microrobot is at Position c, the reference target position and actual position coordinates are (3.5, 2.0, 0.5) and (3.47, 2.00, 0.51), respectively. We calculated the reference distance from the starting position to the reference target position for each step, and calculated the deviation between reference target and predicted position. Accordingly, the path tracking error ratios at Positions c, d, e, f, g, and h, are obtained as shown in Table 5. Because Position b is the initial position of the entire path tracking, there is no error at this time. The error ratios are primarily less than 5%. Finally, the microrobot realized the locomotion along the reference path as shown in Figure 10h. The standard deviation of position is 0.0145 mm. According to the result, we can conclude that the control system based on RBFNN can provide the control direction of each position. Subsequently, the corresponding coil currents can be calculated to generate the rotating magnetic field for driving the robot to move along the reference direction.

In the actual experiment, it is necessary to obtain some training samples for RBFNN learning, to establish the relationship between the reference angle and control angle. First, we can obtain the present position $P_{0}$ of robot. We set the control direction with angle $\gamma_{cont}$ and $\alpha_{cont}$, and calculate the currents of the Helmholtz coils. Thereafter, the Helmholtz coils generate the rotating magnetic field and drive the spring-type robot to the position $P_{ref}$. The simulation result for this case shows that if we want to drive the robot from $P_{0}$ to $P_{ref}$, we can set the control angle $\gamma_{cont}$ and $\alpha_{cont}$ to generate a rotating magnetic field for the movement of the robot. The direction from $P_{0}$ to $P_{ref}$ is the reference direction. We can calculate the angle $\gamma_{ref}$ and $\alpha_{ref}$ of the direction from $P_{0}$ to $P_{ref}$ using Equations (31) and (32). Thus, a training sample with components of $\gamma_{ref}$,$\alpha_{ref}$, $\gamma_{cont}$, and $\alpha_{cont}$ is obtained. In this manner, we can obtain many training samples and train the RBFNN.

After the RBFNN is trained, we can apply the control system based on RBFNN to path tracking control. We can obtain the reference target position and present position of each step, and subsequently calculate the reference angle $\gamma_{ref}$ and $\alpha_{ref}$ to provide as input to the RBFNN. RBFNN outputs the control angle $\gamma_{cont}$ and $\alpha_{cont}$. Next, the control system can derive the current of Helmholtz coils, and subsequently generate the RMF to drive the robot to the reference target position.

## 6. Discussion

Clustering algorithms can classify similar samples into the same cluster, but the conventional clustering algorithms often require the determination of several important parameters based on experience in advance, thus leading to inconvenience. Moreover, when conventional clustering algorithms are applied in some specific situations, the clustering algorithms can be improved to increase efficiency and accuracy. MNNC determines the samples with the highest similarity based on the determination of the nearest neighbors. Because the data of curve fitting and path following control have the characteristics of obvious time or spatial sequence, the performance of the MNNC is considerably improved for this type of data. MNNC reduces the range of determining the nearest neighbor that reduces the computation cost, thereby requiring few parameters to be set. Furthermore, it can adaptively adjust the number of clusters. Moreover, MNNC can determine the abnormal samples in the dataset and adjust them. After the adjusted data is used for curve fitting, the fitting accuracy can be improved by 50%; particularly, the adjustment effect is more prominent when there are not many outliers because the adjustment is performed on the samples with the largest outliers, and the sample adjustment is a gradual process. The abnormal sample adjustment of MNNC can avoid misjudgment and over-adjustment of outliers. For cases with a large number of outliers, the adjustment effect can be enhanced by increasing the number of optimizations.

RBFNN is commonly used in nonlinear systems for curve fitting and path tracking control. Using a clustering algorithm to obtain the initial parameters of RBFNN is a relatively simple method. When MNNC is used to train RBFNN, the number of hidden nodes of RBFNN can be changed by automatically adjusting the number of clusters according to the accuracy requirements of RBFNN, to improve the accuracy of RBFNN. The simulation results show that the curve fitting accuracy of RBFNN trained by MNNC is up to 60% higher than that of other RBFNNs.

When the magnetic microrobot is moving, it is difficult to reach the target position accurately due to the interference of various factors. In this study, the motion mechanism of the magnetic robot is analyzed, and a locomotion control system based on RBFNN is proposed. The system uses RBFNN to determine the reference target of the theoretical locomotion target, and controls the magnetic microrobot to reach the theoretical motion target by moving to the reference target. In order to use MNNC to establish the parameters of RBFNN better, this study enhanced the function of MNNC on the basis of the previous analysis. The simulation results show that the enhanced MNNC demonstrates an improved performance over traditional clustering algorithms in clustering analysis, and there are fewer parameters to be determined in advance. The simulation results show that a better control effect can be obtained after applying RBFNN based on MNNC in the path tracking control of the magnetic robot.

Although MNNC has only been applied in clustering 2D data in this study, it can adapt to multidimensional datasets. This will be verified in future research, and the algorithm will be improved to increase the clustering accuracy and generalization ability.

## 7. Conclusions

A modified nearest neighbor-based clustering algorithm is proposed in this study that does not necessitate the setting of important parameters relying on past experience, and can perform cluster analysis on samples of different densities and shapes. The abnormal samples can be found, and the adjustment of the sample can be realized by this clustering algorithm. The simulation results show that the curve fitting accuracy of the samples optimized by the clustering algorithm is increased by 50%. The number and center of basis functions can be automatically determined by applying this clustering algorithm on the training samples of RBFNN. The simulation results proved that the RBFNN trained in this manner has a higher operating accuracy than the conventional RBFNN; the accuracy in curve fitting is improved by 60%. The simulation result showed that the RBFNN based on the clustering algorithm could improve the accuracy in the path tracking simulation by 20%.

## Figures and Tables

**Table 1:** Clustering index of the clustering results.

Clustering Algorithm	Performance Index	Dataset		
		Data 1	Data 2	Data 3
K-means	ACC	0.3333	0.6683	0.4891
	ARI	0.0233	0.3121	0.3010
DBSCAN	ACC	1	1	0.8558
	ARI	1	1	0.9019
MNNC	ACC	1	1	0.9994
	ARI	1	1	0.9994

**Table 2:** Errors of curve fitting with/without the adjustment of abnormal samples.

Curve Type	2D Curve		3D Curve
Noise style	Randomly distributed	Six points with noise	Randomly distributed
Errors without adjustment	3.0793	2.7281	516.6542
Errors with adjustment	2.8145	1.3292	485.3374

**Table 3:** Test results of RBFNN based on different clustering algorithms.

Algorithm	Cluster Number	Training Error	Test Error
K-means	59	2.27°	2.89°
DBSCAN	59	2.22°	2.24°
MNNC	59	2.10°	2.21°

**Table 4:** Coil current equation corresponding to Figure 10b–h.

Position	$I_{x}(A)$	$I_{y}(A)$	$I_{z}(A)$
b	$I_{x}=0.999\sin(360t+90.69{}^{\circ})$	$I_{y}=0.238\sin(360t+12.72{}^{\circ})$	$I_{z}=-0.973\sin(360t)$
c	$I_{x}=0.909\sin(360t+95.96{}^{\circ})$	$I_{y}=0.471\sin(360t+64.89{}^{\circ})$	$I_{z}=-0.975\sin(360t)$
d	$I_{x}=0.592\sin(360t+109.57)$	$I_{y}=0.84\sin(360t+80.86{}^{\circ})$	$I_{z}=-0.971\sin(360t)$
e	$I_{x}=0.234\sin(360t+160.18{}^{\circ})$	$I_{y}=0.997\sin(360t+89{}^{\circ})$	$I_{z}=-0.975\sin(360t)$
f	$I_{x}=0.481\sin(360t+245.64{}^{\circ})$	$I_{y}=0.904\sin(360t+96.15{}^{\circ})$	$I_{z}=-0.975\sin(360t)$
g	$I_{x}=0.838\sin(360t+261.24{}^{\circ})$	$I_{y}=0.592\sin(360t+108.55{}^{\circ})$	$I_{z}=-0.974\sin(360t)$
h	$I_{x}=0.997\sin(360t+268.84{}^{\circ})$	$I_{y}=0.272\sin(360t+163.4{}^{\circ})$	$I_{z}=-0.965\sin(360t)$

*t* is time (s).

**Table 5:** Path tracking error ratio of position b–h.

Position	Reference Angle (°)		Predicted Angle (°)		Angle Error Ratio (%)		Reference Coordinate (mm)	Predicted Coordinate (mm)	Position Error Ratio (%)
	$\alpha_{ref}$	$\gamma_{ref}$	$\alpha_{pre}$	$\gamma_{pre}$	γ	α			
b	93	76.57	93	76.57			(4, 0, 0)	(4, 0, 0)	0
c	117	76.57	115.78	75.79	1.04%	1.02%	(3.46, 2.0, 0.5)	(3.47, 2.00, 0.51)	2.48%
d	147	76.57	144.40	79.89	1.77%	4.34%	(2.0, 3.46, 1.0)	(2.01, 3.48, 0.98)	7.31%
e	177	76.57	172.34	77.90	2.63%	1.74%	(0, 4.0, 1.5)	(−0.00, 4.03, 1.49)	8.26%
f	207	76.56	208.69	76.30	0.81%	0.35%	(−2.0, 3.46, 2.0)	(−1.99, 3.45, 2.00)	2.90%
g	237	76.57	237.56	76.97	0.24%	0.53%	(−3.46, 2.0, 2.5)	(−3.46, 1.99, 2.49)	1.19%
h	267	76.56	269.61	76.39	0.98%	0.23%	(−4, 0, 3)	(−3.98, 0.01, 3.01)	4.44%

## Data Availability

Data can be requested from the corresponding authors.

## Appendix A

**Table A1:** List of acronyms.

RBFNN	Radial basis function neural network
MNNC	Modified nearest neighbor-based clustering
PID	Proportion integral differential
SVM	Support vector machines
GA	Genetic algorithm
AI	Artificial immune
DBSCAN	Density-based spatial clustering of applications with noise
DPC	Density peaks clustering
ACC	Accuracy
ADI	Adjusted Rand index
RMF	Rotating magnetic field
BIRCH	Balanced iterative reducing and clustering using hierarchies
CLIQUE	Clustering in QUEst

## Appendix B

**Table A2:** List of variables.

*d_i_*	Distance between sample and its nearest neighbor
*H*	The number of d_i_ level
*P_i_*	Sample
ΔD	Distance change
*d_step_*	Distance step
*D*	Total distances between samples and centroid of cluster before merged
*D’*	Total distances between samples and centroid of cluster after merged
*Quan_i_*	Quantity of samples of cluster i
C	Center of cluster
*R*	Scanning radius of DBSCAN
*T_m_*	Magnetic torque
*V*	Volume of microrobot
*M*	Magnetization of microrobot
*B*	External magnetic field
*γ*	Polar angle
α	Azimuthal angle
*n_B_*	Normal vector of plan P
*P_cont_*	Control position
*P_ref_*	Reference position
*P_act_*	Actual position
*P_guid_*	Guidance position
*P_pre_*	Predicted position
*γ_ref_*	Reference polar angle
*α_ref_*	Reference azimuthal angle
*γ_cont_*	Control polar angle
*α_cont_*	Control azimuthal angle
*γ_guid_*	Guidance polar angle
*α_guid_*	Guidance azimuthal angle
*γ_pre_*	Predicted polar angle
*α_pre_*	Predicted azimuthal angle
*γ_comp_*	Compensation of polar angle
*α_comp_*	Compensation of azimuthal angle
*d_cont_*	Control direction
*d_guid_*	Guidance direction
*d_ref_*	Reference direction
*d_pre_*	Predicted direction
*d_act_*	Actual direction
*Q_i_*	Output of hidden layer neuron of RBFNN
*c_i_*	Center of hidden layer neuron of RBFNN
*w_i_*	Weight between hidden layer and output layer of RBFNN
*σ*	Width of hidden layer neuron of RBFNN
*μ_0_*	Permeability of free space
*N*	Turns number of coil
*K_B_*	Magnetic field coefficient of Helmholtz coil
*I*	Coil current
*I_x_*	Coil current of *x*-axis
*I_y_*	Coil current of *y*-axis
*I_z_*	Coil current of *z*-axis
*err_gamma_*	Error of polar angle
*err_alpha_*	Error of azimuthal angle
*err_position_*	Error of position
